# AI-Assisted Scoring Improves Interobserver Agreement in Breast Cancer Biomarker Evaluation

**DOI:** 10.3390/cancers18183003

**Published:** 2026-09-16

**Authors:** Wiebke Solass, Clara Desteffani, Florin Kalberer, Ben Scattolo, Branislav Zagrapan, Donna Mulkern, Peter Caie, Martin Wartenberg, Inti Zlobec, Meredith Lodge

**Affiliations:** 1 Institute of Tissue Medicine and Pathology (ITMP), University of Bern, 3008 Bern, Switzerlandbranislav.zagrapan@unibe.ch (B.Z.);; 2 Indica Labs, Albuquerque, NM 87109, USAmlodge@indicalab.com (M.L.); 3 Department Digital Medicine, University of Bern, 3008 Bern, Switzerland

**Keywords:** breast cancer, digital pathology, artificial intelligence, biomarker assessment, immunohistochemistry, AI-assisted diagnosis, HER2, Ki67, estrogen receptor, progesterone receptor

## Abstract

**Background:** Immunohistochemical assessment of estrogen receptor (ER), progesterone receptor (PR), proliferation marker Ki67, and human epidermal growth factor receptor 2 (HER2) represents a central component of the diagnostic workflow in invasive breast carcinoma, directly influencing therapeutic decision-making, prognosis, and patient management. In this study, we evaluated the use of Indica Labs’ clinical image analysis platform for AI-assisted breast cancer biomarker scoring in a prospective observer study conducted at the University of Bern. The study compared biomarker assessment by novice observers and trained pathologists with and without AI assistance, with a focus on interobserver agreement, scoring consistency, and the potential role of AI-assisted workflows in diagnostic practice and training environments. **Methods:** Fifty breast cancer cases comprising 250 immunohistochemically stained slides for ER, PR, HER2, and Ki67 were independently assessed by three medical students and two board-certified pathologists in two sequential evaluation rounds. In the first round, biomarkers were scored using conventional digital pathology assessment without AI support (Visual Dx). Following a four-week washout period, the same cases were re-evaluated with assistance from HALO Breast IHC AI (AI-Assisted Dx). Observer agreement and scoring concordance were analyzed to compare performance between novice observers and experienced pathologists under unassisted and AI-assisted conditions. **Results:** AI-assisted assessment increased interobserver agreement at clinically relevant cutoffs across all four biomarkers in both novice observers and pathologists. Similarly, Fleiss’ kappa values improved for all biomarkers following AI assistance, with an average increase of 0.42 across observer groups. When all participants were analyzed collectively, agreement at clinical decision thresholds also improved, indicating greater concordance between novice observers and experienced pathologists during AI-assisted evaluation. **Conclusions:** HALO Breast IHC AI supported both novice observers and experienced pathologists by improving scoring concordance and interobserver agreement across clinically relevant breast cancer biomarkers. These findings suggest that AI-assisted workflows may contribute to greater standardization and reproducibility in biomarker assessment and may also provide supportive value in educational and training settings.

## Simple Summary

Breast cancer treatment decisions often depend on measuring specific proteins in tumour tissue, a process usually performed by experts examining microscope images. This assessment can vary between observers, particularly among less experienced staff, which may affect diagnostic consistency. This study investigated whether an artificial intelligence tool could help medical students and experienced pathologists score these tumour markers more consistently. Fifty breast cancer cases were evaluated twice: first using standard visual assessment, then again with computer assistance four weeks later. Agreement between observers improved substantially when the computer tool was used, for both trainees and experienced pathologists, bringing their results closer together. These findings suggest that computer-assisted scoring could help make breast cancer diagnosis more consistent across observers with different experience levels, which may be valuable in training settings and in laboratories with fewer specialist staff. Larger, multi-center studies are needed before this approach can be adopted in routine clinical practice.

## 1. Introduction

Accurate and reproducible assessment of immunohistochemical (IHC) biomarkers, including estrogen receptor (ER), progesterone receptor (PR), human epidermal growth factor receptor 2 (HER2), and the proliferation marker Ki67, is essential for the diagnosis, prognostication, and therapeutic management of invasive breast carcinoma. These biomarkers directly influence treatment decisions, including the use of endocrine therapy, anti-HER2 targeted agents, and chemotherapy, and are routinely evaluated in diagnostic pathology practice according to established guidelines such as those from the American Society of Clinical Oncology and the College of American Pathologists (ASCO/CAP) [1,2,3].

Despite their clinical importance, interpretation of breast cancer IHC biomarkers remains susceptible to inter- and intra-observer variability, particularly in cases with borderline, heterogeneous, or low-level expression patterns [4,5,6]. Variability in interpretation may be more pronounced among less experienced observers who have limited exposure to histopathological assessment and biomarker scoring [7,8,9]. With the increasing adoption of digital pathology workflows, computational image analysis tools have emerged as potential approaches to improve scoring consistency and support diagnostic assessment.

Recent advances in computational pathology have enabled the development of artificial intelligence (AI)-assisted systems for automated and semi-automated evaluation of breast cancer biomarkers. Previous studies have demonstrated high concordance between AI-assisted image analysis platforms and expert pathologist assessment for ER, PR, HER2, and Ki67 scoring, supporting their potential role in improving reproducibility and standardization in breast cancer diagnostics [10,11,12,13,14,15]. Such systems may provide objective quantification and reduce observer-related variability during biomarker interpretation.

In addition to their diagnostic applications, there is increasing interest in understanding how AI-assisted pathology tools influence observer performance and decision-making processes. By providing quantitative support, visual overlays, and standardized assessment frameworks, these systems may facilitate more consistent scoring behaviour among users with varying levels of experience [16,17]. However, the extent to which AI assistance supports interpretation, influences observer concordance, or contributes to educational settings remains incompletely understood.

Beyond biomarker-specific AI scoring, emerging imaging modalities—including optical coherence tomography, Raman spectroscopy, photoacoustic imaging, hyperspectral imaging, contrast-enhanced spectral mammography, and multispectral imaging—are also being investigated as adjuncts to breast cancer diagnosis, generally reporting improved sensitivity and specificity over conventional methods, although they remain complementary to, rather than replacements for, morphological and immunohistochemical assessment [18]. Consistent with the present findings, a recent meta-analysis of hyperspectral-imaging-based computer-aided detection of breast cancer reported average sensitivity, specificity, and accuracy of 78%, 89%, and 87%, respectively, across eight studies [19], reinforcing the broader trend toward AI-assisted approaches in breast cancer imaging and diagnostics.

In the present study, we evaluated the impact of HALO Breast IHC AI (Indica Labs, Albuquerque, NM, USA) on the assessment of ER, PR, HER2, and Ki67 in invasive breast carcinoma using a prospective observer study design. Three undergraduate medical students without prior pathology training and two board-certified pathologists independently scored digitized breast cancer cases first without AI assistance and subsequently with AI-assisted support following a washout interval. We assessed interobserver agreement and concordance at clinically relevant thresholds to determine whether AI assistance improved scoring consistency across observers with different levels of experience. We hypothesized that AI-assisted assessment would improve observer concordance and support more standardized biomarker evaluation.

In contrast to prior work, which has largely examined AI-assisted concordance with expert assessment in isolation, this study directly compares novice and expert observers scoring the same case set under both unassisted and AI-assisted conditions, allowing the differential impact of AI assistance across experience levels to be assessed within a single prospective cohort. This design distinguishes the present study from previous reports and provides evidence specifically relevant to the potential role of AI-assisted scoring in supporting less experienced observers.

## 2. Methods

### 2.1. Study Design

This prospective observer study included two rounds of immunohistochemical (IHC) biomarker assessment performed independently by three undergraduate medical students without prior pathology training and two board-certified breast pathologists. All participants evaluated the same set of digitized breast cancer slides under standardized conditions. The study is prospective with respect to the observer scoring workflow, which was defined and conducted according to a pre-specified protocol; the underlying tissue cases were retrospectively identified from the ITMP archive, as described under Section 2.3.

Prior to case evaluation, the medical students underwent structured introductory training delivered by a consultant pathologist. The training consisted of a 2 h session covering the ASCO/CAP guidelines for breast biomarker assessment and relevant concepts from the WHO Classification of Breast Tumours (5th edition) [20]. Training topics included interpretation criteria and positivity thresholds for ER, PR, HER2, and Ki67, identification of invasive carcinoma versus ductal carcinoma in situ (DCIS), and recognition of potential false-positive staining in benign epithelial structures. Participants were additionally provided with a written scoring reference guide. The board-certified breast pathologists did not receive this introductory pathology training, as breast biomarker assessment formed part of their routine diagnostic practice. Both the medical students and pathologists subsequently completed separate software training sessions delivered by an Indica Labs subject matter expert, which covered navigation of the HALO AP^®^ digital pathology platform (Indica Labs, Albuquerque, NM, USA), biomarker scoring workflows, and use of the HALO Breast IHC AI application. No additional pathology training or retraining was provided during the four-week washout period between assessment rounds. Following this interval, participants reassessed the study cases using AI-assisted scoring support as described below.

**Figure 1:**
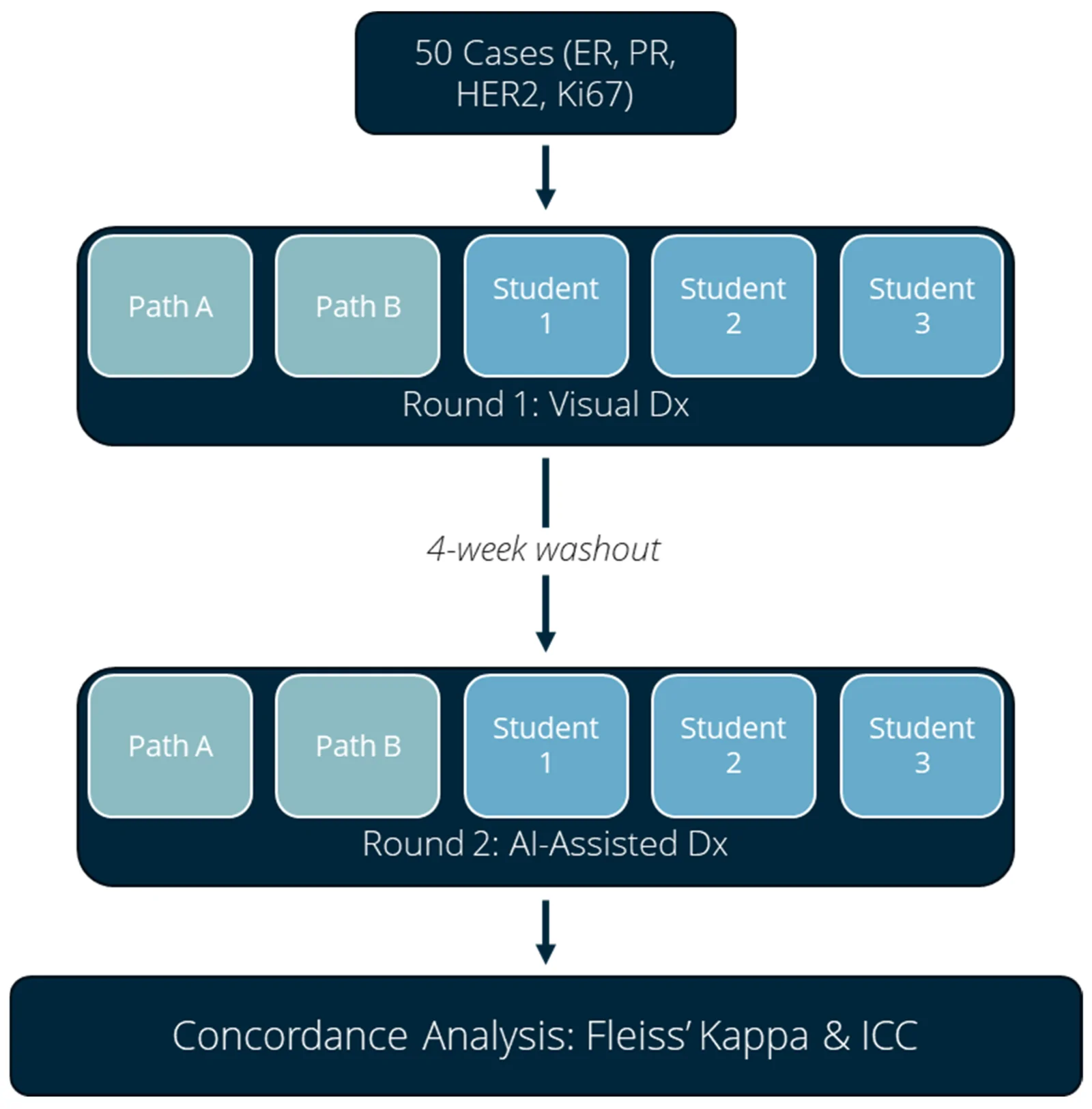
Study design.

In the first evaluation round, all observers performed visual assessment via a digital pathology platform, without AI assistance (Visual Dx). Following a four-week washout interval intended to reduce recall bias, the same cases were reassessed using HALO Breast IHC AI-assisted scoring support (AI-Assisted Dx) (Figure 1). During the AI-assisted round, observers retained responsibility for the final interpretation and scoring decisions. The same study workflow was applied to both pathologists and novice observers.

Fifty primary invasive breast carcinoma cases stained for ER, PR, HER2, and Ki67 were independently evaluated by two board-certified pathologists and three undergraduate medical students across two assessment rounds. In Round 1, all observers performed digital visual assessment without AI support (Visual Dx). Following a four-week washout interval, the same cases were reassessed using HALO Breast IHC AI-assisted evaluation (AI-Assisted Dx). Interobserver agreement and concordance between assessment rounds were analyzed using Fleiss’ kappa and intraclass correlation coefficient (ICC) metrics.

### 2.2. Digital Pathology Platform

All slide assessments were performed using the HALO AP^®^ digital pathology platform (Indica Labs, Albuquerque, NM, USA). AI-assisted evaluation was conducted using HALO Breast IHC AI, a clinically validated deep learning-based image analysis tool designed for quantitative assessment of breast cancer biomarkers. The platform incorporates multiple classification models to identify invasive tumour regions, exclude non-relevant tissue components and artefacts, and generate cell-level quantification of ER, PR, HER2, and Ki67 staining in accordance with established clinical scoring guidelines.

### 2.3. Study Samples

Fifty previously diagnosed invasive breast carcinoma cases comprising 250 digitized slides, including hematoxylin and eosin (H&E)-stained sections and corresponding immunohistochemical preparations, were retrospectively selected from the archives of the Institute of Tissue Medicine and Pathology (ITMP), University of Bern. Case selection and anonymization were performed by the Translational Research Unit (TRU) in accordance with institutional ethical and data protection requirements. Cases were selected sequentially to reflect the range of histological subtypes and biomarker expression profiles encountered in routine diagnostic practice. Consecutive eligible cases meeting the inclusion criteria below were selected without prior knowledge of, or selection based on, biomarker status, diagnostic outcome, or scoring difficulty. Available clinicopathological data included tumour diagnosis, histological grade, and routine clinical IHC assessments for ER, PR, HER2, and Ki67 (Table 1). These routine clinical IHC assessments correspond to the original diagnostic score issued in each case’s signed clinical pathology report at ITMP, which, in accordance with standard dual-reporting practice, is signed by two pathologists. This original clinical score was used as the reference value for the comparisons; no re-evaluation or adjudication of the original clinical score was performed as part of the present study.

Whole-slide images were acquired using a Hamamatsu NanoZoomer S360 scanner (Hamamatsu Photonics, Hamamatsu, Japan) at ×40 magnification (0.23 μm/pixel resolution). Immunohistochemical staining was performed using the following antibodies: ER (SP1, Ventana/Roche Diagnostics, Oro Valley, AZ, USA), PR (16 + SAN27, Novocastra/Leica Biosystems, Newcastle upon Tyne, UK), Ki67 (SP6, Cell Marque/MilliporeSigma, Rocklin, CA, USA), and HER2 (4B5, PATHWAY, Ventana/Roche Diagnostics). Cases were included if slides demonstrated sufficient invasive tumour tissue and adequate staining quality for biomarker assessment. Exclusion criteria included insufficient tumour content, suboptimal staining quality, or the presence of significant technical artefacts.

### 2.4. Scoring Procedure and AI Integration

In the initial assessment round (Visual Dx), all whole-slide images were independently evaluated using conventional digital pathology interpretation without AI assistance. Observers assessed biomarker expression according to established clinical criteria, including staining intensity and the proportion of positively stained invasive tumour cells.

**Figure 2:**
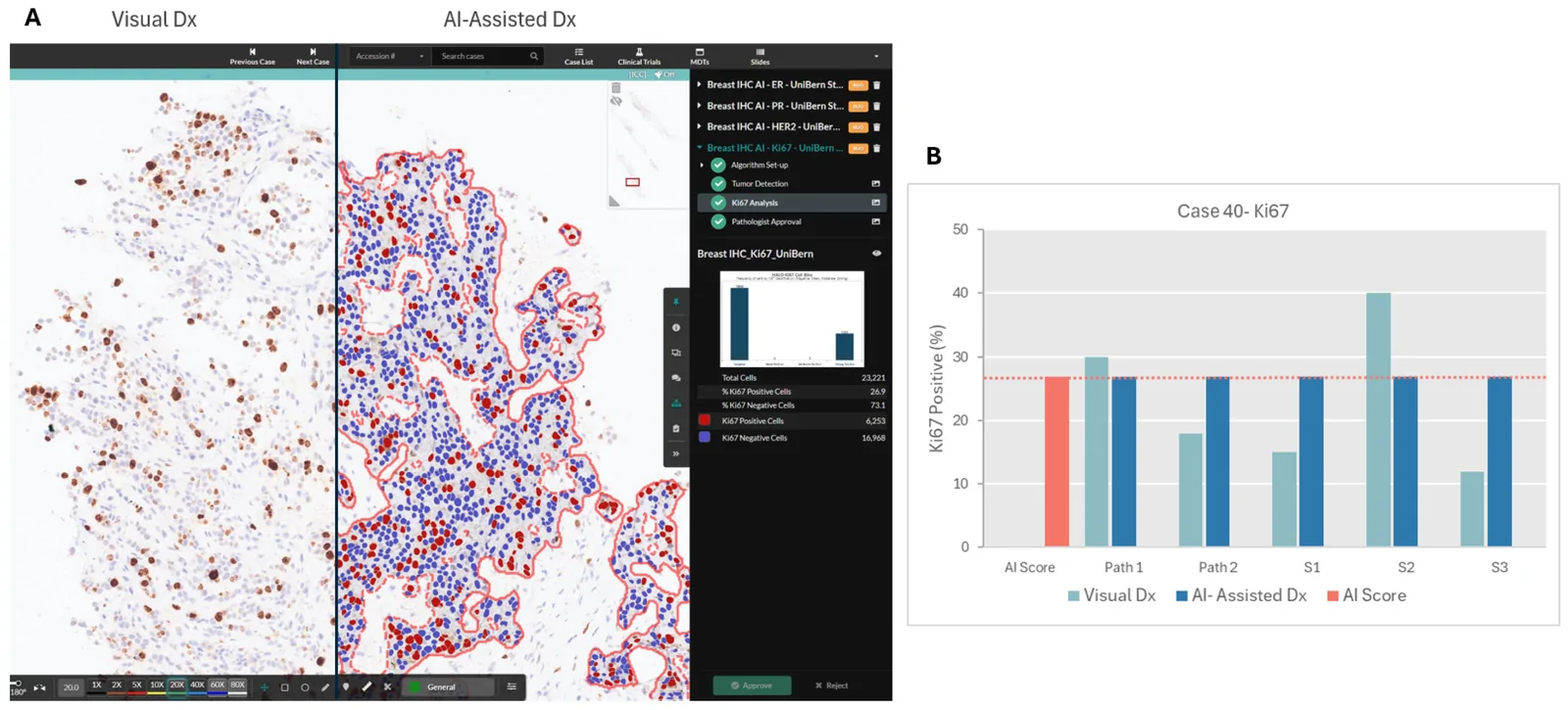
Example of AI-assisted Ki67 assessment and its influence on observer concordance in a representative case (Case 40): (**A**) Representative comparison between conventional digital visual assessment (Visual Dx, left) and AI-assisted assessment (AI-Assisted Dx, right) for Ki67 immunohistochemistry. In the AI-assisted output, invasive tumour regions are identified by the algorithm, while Ki67-positive nuclei are highlighted in red and Ki67-negative nuclei in blue. The platform additionally provides quantitative outputs, including total cell counts and the proportion of positive and negative tumour cells. (**B**) Comparison of Ki67 scores (%) assigned by the AI system (red), two board-certified pathologists (Path 1–2), and three medical students (S1–S3) during conventional visual assessment (light blue) and AI-assisted assessment (dark blue). The dashed red line indicates the AI-derived score. AI-assisted evaluation was associated with reduced variability and greater concordance among observers in this representative example.

Following the washout interval, the same slides were reassessed using HALO Breast IHC AI-assisted evaluation (AI-Assisted Dx). During this second round, the AI platform provided quantitative biomarker outputs together with visual guidance tools, including tumour classification overlays, cell-level annotations, and numerical scoring summaries (Figure 2). Observers retained responsibility for reviewing the AI-generated outputs and determining the final reported scores.

All biomarker assessments were performed according to established ASCO/CAP guidelines for ER, PR, and HER2 interpretation. Ki67 was quantified as the percentage of positively stained invasive tumour nuclei, with a >15% threshold applied for categorical classification [21,22,23]. Observers were blinded to their previous assessments during the second evaluation round to minimize recall bias. AI-assisted results were subsequently compared across observer groups to evaluate changes in interobserver agreement and scoring concordance.

### 2.5. Statistical Analysis

Across both assessment rounds, the five observers evaluated a total of 200 slides, generating 2000 individual scoring observations. Cases with incomplete or missing scores were excluded from the corresponding analyses (Supplementary Table S1). Briefly, the number of slides excluded (combined across students and pathologists) was, for ICC analyses: ER, 3 in Visual Dx and 3 in AI-Assisted Dx; PR, 4 and 4; Ki67, 4 and 0; and for Fleiss’ kappa analyses: ER, 2 and 3; PR, 4 and 1; Ki67, 4 and 0; and HER2, 4 and 1 (Supplementary Table S1).

The primary study endpoints were interobserver agreement and scoring concordance between conventional visual assessment (Visual Dx) and AI-assisted assessment (AI-Assisted Dx). Intraclass correlation coefficients (ICCs) were used to assess agreement for continuous variables, calculated using a two-way random-effects, absolute-agreement, single-rater model (ICC(2,1)), as all observers scored the same fixed set of cases and were treated as a random sample from a larger population of potential raters, while Fleiss’ kappa statistics were applied to categorical classifications based on clinically relevant biomarker thresholds; kappa estimates are reported with associated *p*-values (testing the null hypothesis of no agreement beyond chance), as the R package used for these analyses does not provide 95% confidence intervals for Fleiss’ kappa. Percentage agreement was additionally calculated as the proportion of concordant classifications among observers. Concordance between AI-derived scores and routine clinical assessment data from the Institute of Tissue Medicine and Pathology (ITMP) was also evaluated.

To standardize comparisons across observers and AI outputs, continuous AI-derived values were rounded to the nearest whole number prior to threshold-based categorical classification. ICC estimates are reported as point values for each biomarker and observer group. Statistical analyses were performed using R software (version 4.6.1) [24], including the irr package for agreement analysis [25] and the ordinal package for mixed-effects model analysis [26].

## 3. Results

**Figure 3:**
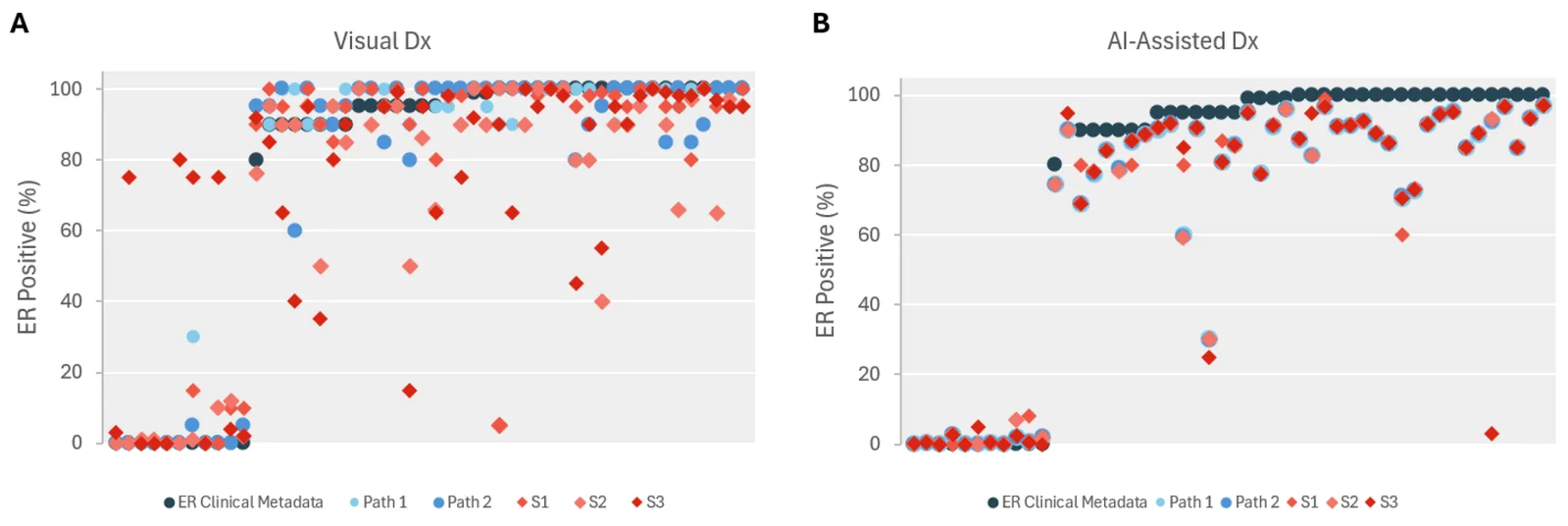
ER scoring during conventional visual assessment and AI-assisted assessment. Scatter plots showing ER percentage positivity scores assigned by pathologists, medical students, and routine clinical assessment data during conventional visual assessment (Visual Dx, (**A**)) and AI-assisted assessment (AI-Assisted Dx, (**B**)). Each data point represents an individual observer score for one of the 50 breast cancer cases. Cases are displayed along the *x*-axis and ordered according to ER positivity levels derived from routine clinical metadata.

**Figure 4:**
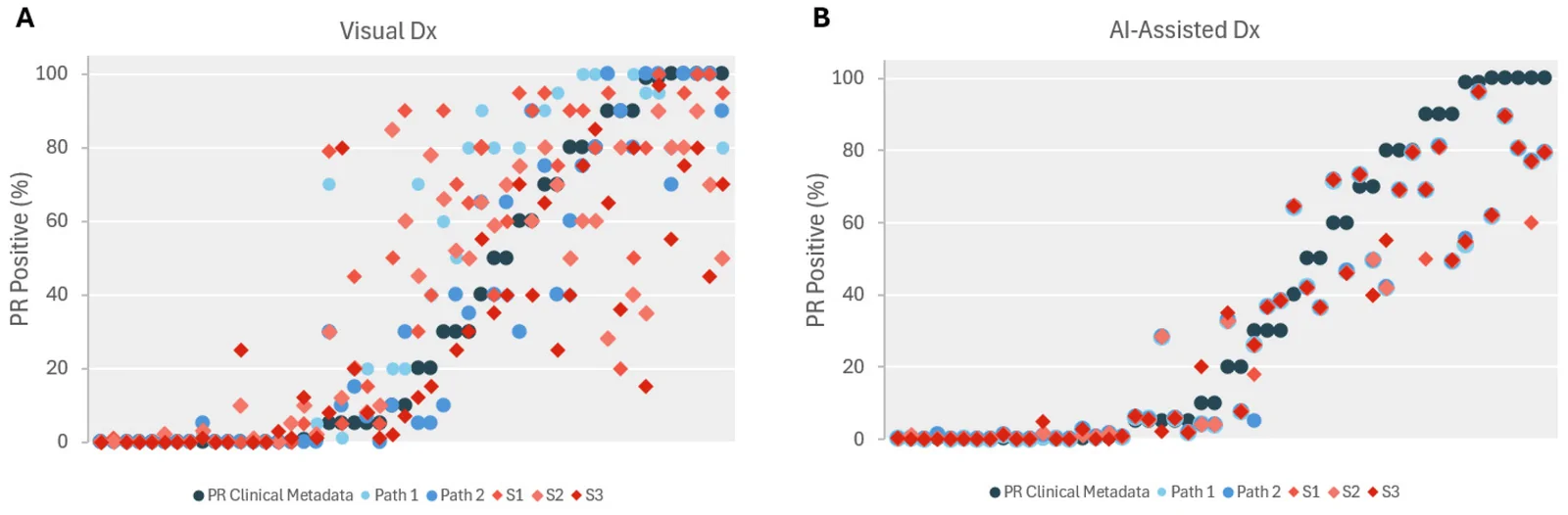
PR scoring during conventional visual assessment and AI-assisted assessment. Scatter plots showing PR percentage positivity scores assigned by pathologists, medical students, and routine clinical assessment data during conventional visual assessment (Visual Dx, (**A**)) and AI-assisted assessment (AI-Assisted Dx, (**B**)). Each data point represents an individual observer score for one of the 50 breast cancer cases. Cases are displayed along the *x*-axis and ordered according to PR positivity levels derived from routine clinical metadata.

**Figure 5:**
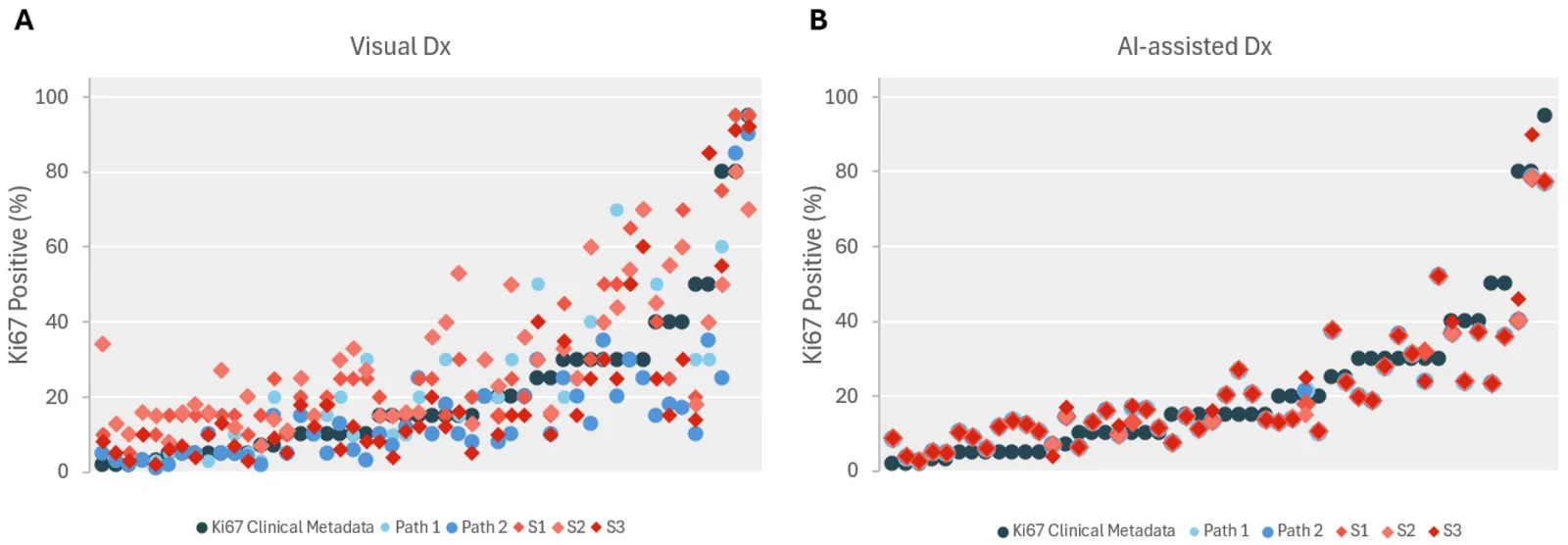
Ki67 scoring during conventional visual assessment and AI-assisted assessment. Scatter plots showing Ki67 percentage positivity scores assigned by pathologists, medical students, and routine clinical assessment data during conventional visual assessment (Visual Dx, (**A**)) and AI-assisted assessment (AI-Assisted Dx, (**B**)). Each data point represents an individual observer score for one of the 50 breast cancer cases. Cases are displayed along the *x*-axis and ordered according to Ki67 positivity levels derived from routine clinical metadata.

**Figure 6:**
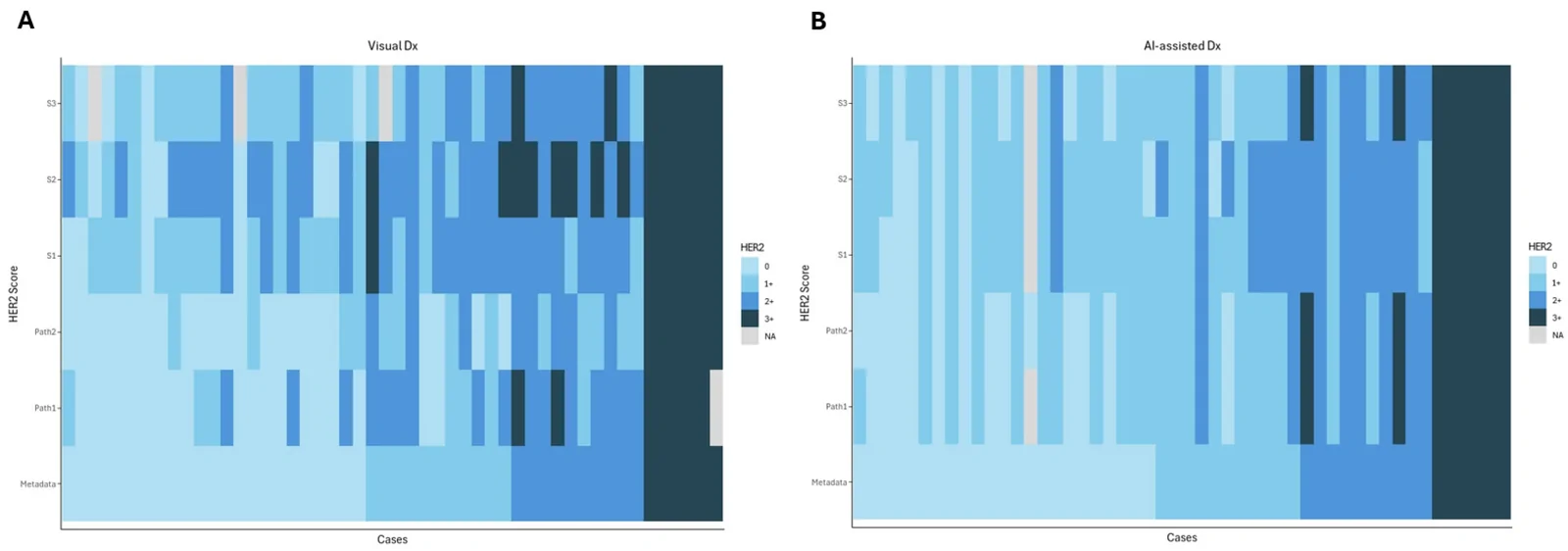
HER2 scoring during conventional visual assessment and AI-assisted assessment. Heat maps showing categorical ASCO/CAP HER2 scores assigned by pathologists, medical students, and routine clinical assessment data during conventional visual assessment (Visual Dx, (**A**)) and AI-assisted assessment (AI-Assisted Dx, (**B**)). Each data point represents an individual observer classification for one of the 49 evaluable breast cancer cases. Grey fields (NA) indicate missing scores. Cases are displayed along the *x*-axis and ordered according to HER2 classification derived from routine clinical metadata.

AI-assisted assessment was associated with improved interobserver agreement and scoring concordance across all evaluated biomarkers (Figure 3, Figure 4, Figure 5 and Figure 6). Both novice observers and pathologists demonstrated increases in ICC values, Fleiss’ kappa statistics, and percentage agreement during the AI-assisted assessment round compared with conventional visual evaluation. Improvements were most pronounced among novice observers, with reduced interobserver variability and greater concordance with experienced pathologist assessments.

Overall, AI-assisted scoring contributed to more consistent biomarker interpretation across observers with differing levels of experience, supporting the potential role of computational pathology tools in standardized IHC assessment workflows.

### 3.1. AI Discrepancies and Clinical Impact

During the AI-assisted assessment round, no cases were identified in which the AI-assisted classification was discordant with the consensus interpretation of both pathologists and novice observers. Complete concordance among AI-assisted assessments, pathologist evaluations, and novice observer classifications was observed in 92% of ER and PR cases, 46% of Ki67 cases, and 80% of HER2 cases.

Across all biomarkers, 26 discordant instances were identified between AI-derived classifications and routine clinical metadata. The highest level of disagreement was observed for Ki67 assessments near the applied >15% classification threshold, reflecting the known variability associated with Ki67 interpretation in breast cancer pathology. Detailed agreement and discordance metrics are summarized in Table 2. As the routine clinical metadata used in this comparison represents the original, non-adjudicated clinical report rather than an independently established reference standard, the values in Table 2 should be interpreted as a measure of concordance with the existing clinical record rather than as a formal diagnostic accuracy metric.

### 3.2. Impact of AI Assistance on ER Scoring

AI-assisted assessment was associated with improved interobserver agreement for ER scoring among both novice observers and pathologists. Among medical students, the intraclass correlation coefficient (ICC) increased from 0.76 during conventional visual assessment to 0.95 during AI-assisted evaluation. Pathologist ICC values increased from 0.97 to 1.00 following AI assistance (Table 3).

Using the clinically relevant 1% positivity threshold, Fleiss’ kappa values for student assessments increased from 0.64 to 0.80 between the Visual Dx and AI-Assisted Dx rounds, while pathologist agreement increased from 0.94 to 1.00 (Table 4). Percentage agreement among students improved from 85.4% to 91.5% with AI assistance, whereas pathologists demonstrated complete agreement during the AI-assisted assessment round.

### 3.3. Impact of AI Assistance on PR Scoring

AI-assisted assessment was associated with substantial improvements in interobserver agreement for PR scoring among both novice observers and pathologists. Among medical students, the intraclass correlation coefficient (ICC) increased from 0.70 during conventional visual assessment to 0.99 during AI-assisted evaluation. Pathologist ICC values similarly increased from 0.86 to 1.00 following AI assistance (Table 3).

Using the clinically relevant 1% positivity threshold, Fleiss’ kappa values for student assessments increased from 0.17 during Visual Dx to 0.81 during AI-Assisted Dx, while pathologist agreement increased from 0.33 to 0.89 (Table 4). Percentage agreement among students improved from 18.8% to 73.5%, whereas agreement among pathologists increased from 43.8% to 90% following AI-assisted assessment.

### 3.4. Impact of AI Assistance on Ki67 Scoring

AI-assisted assessment was associated with improved interobserver agreement for Ki67 scoring among both novice observers and pathologists. Among medical students, the intraclass correlation coefficient (ICC) increased from 0.78 during conventional visual assessment to 0.99 during AI-assisted evaluation. Pathologist ICC values similarly increased from 0.74 to 1.00 following AI assistance (Table 3).

Using the predefined 15% positivity threshold, Fleiss’ kappa values for student assessments increased from 0.18 to 0.92 between the Visual Dx and AI-Assisted Dx rounds, while pathologist agreement increased from 0.58 to 1.00 (Table 4). Percentage agreement among students improved from 50% to 94%, whereas pathologist agreement increased from 79% to 100% during AI-assisted assessment.

### 3.5. Impact of AI Assistance on HER2 Scoring

HER2 assessment was categorized according to current ASCO/CAP guidelines into negative (0/1+), equivocal (2+), and positive (3+) groups. AI-assisted assessment was associated with improved agreement among both novice observers and pathologists. Fleiss’ kappa values for student assessments increased from 0.44 during conventional visual assessment to 0.78 during AI-assisted evaluation, while pathologist agreement increased from 0.46 to 1.00 (Table 4).

Percentage agreement among students improved from 46.8% to 81.6% following AI assistance, whereas agreement among pathologists increased from 71.4% to 100%. Improvements in observer concordance were particularly evident in cases with borderline HER2 expression patterns, including 1+ and 2+ classifications.

### 3.6. Mixed-Effects Modelling of Categorical Biomarker Scores

Mixed-effects modelling of categorical biomarker scores demonstrated patterns consistent with the observed improvements in interobserver agreement. Across biomarkers, cumulative link mixed models showed that AI-assisted assessment was associated with reduced differences between student and pathologist ratings, with evidence of convergence in scoring behaviour during the AI-assisted round (Table 5). For ER, a positive group-by-round interaction (β = 1.40) indicated that baseline differences between students and experts were attenuated following AI-assisted evaluation, consistent with increased agreement between observer groups. Similar interaction patterns were observed across PR, Ki67, and HER2, where the effects of AI-assisted scoring in students partially or fully offset baseline group differences, suggesting a greater relative shift in scoring among novice observers compared with experienced pathologists. However, the magnitude and statistical significance of these effects varied by biomarker, and not all interaction terms reached statistical significance, particularly for ER. Specifically, the student × AI-assisted interaction for ER (β = 1.40, *p* = 0.220) and the AI-Assisted-versus-Visual main effect for HER2 (β = 0.61, *p* = 0.077) did not reach statistical significance, and these non-significant effects should not be interpreted as evidence of a robust group-convergence effect for these specific terms. Overall, these findings support a consistent trend towards alignment of student and expert scoring during AI-assisted assessment, with a more pronounced influence of AI on student scoring behaviour, in keeping with the observed improvements in interobserver concordance reported across agreement metrics.

## 4. Discussion

This prospective observer study demonstrates that AI-assisted immunohistochemical (IHC) assessment was associated with improved scoring concordance and reduced interobserver variability across clinically relevant breast cancer biomarkers: ER, PR, HER2, and Ki67. Improvements were observed among both novice observers and experienced pathologists, with the largest gains seen for biomarkers that are known to exhibit substantial interpretive variability, particularly PR, HER2, and Ki67. These findings support the potential role of computational pathology tools as adjunctive decision-support systems that may contribute to more standardized biomarker assessment workflows in breast cancer pathology.

Among the evaluated biomarkers, PR and Ki67 demonstrated particularly pronounced improvements in observer agreement during AI-assisted assessment. PR interpretation can be challenging in cases with heterogeneous staining patterns or admixed benign epithelium, while Ki67 scoring is well recognized for its substantial interobserver variability in routine practice [1,27,28]. In the present study, student Fleiss’ kappa values increased from 0.17 to 0.81 for PR assessment and from 0.18 to 0.92 for Ki67 assessment following AI assistance. Similar improvements were also observed among pathologists. These findings are consistent with previous studies demonstrating that digital image analysis platforms can improve reproducibility for biomarkers that are difficult to assess manually. Acs et al. reported improved inter-platform and interobserver agreement for Ki67 scoring using digital image analysis approaches [10], while Koopman et al. demonstrated robust concordance between automated and manual Ki67 scoring using virtual dual staining workflows [13].

HER2 assessment, particularly in borderline 1+ and 2+ cases, also showed improved observer concordance during AI-assisted evaluation. HER2 interpretation is known to be susceptible to variability due to incomplete membrane staining, weak intensity patterns, and subjective threshold interpretation. Our findings align with prior reports by Jung et al., who demonstrated improved interobserver agreement for HER2, ER, and PR scoring using AI-assisted image analysis in breast cancer cohorts [29]. Similarly, Holten-Rossing et al. showed that automated HER2 analysis improved scoring reproducibility compared with conventional microscopy-based evaluation [12]. Although HER2-low and HER2-ultralow classifications were not incorporated into the present study design due to evolving clinical definitions at the time of data acquisition, these emerging therapeutic categories represent an important area for future investigation using AI-assisted scoring approaches.

The observed improvements in observer concordance are consistent with broader developments in computational pathology across multiple tumour types. Previous studies have demonstrated that AI-assisted scoring systems can improve reproducibility and standardization for biomarker interpretation in esophageal and lung cancers [30,31]. Together, these findings support the growing role of AI-assisted image analysis as a complementary tool within digital pathology workflows rather than as a replacement for expert interpretation.

Importantly, the present study also highlights several considerations regarding human–AI interaction in diagnostic assessment. During the AI-assisted evaluation round, observer scores demonstrated substantial convergence, particularly among novice participants. These findings are supported by mixed-effects modelling of categorical scores, which demonstrated a relative reduction in differences between student and pathologist ratings during AI-assisted assessment. While this likely reflects improved standardization and quantitative support provided by the AI platform, an anchoring effect toward AI-generated outputs cannot be excluded. Observers retained responsibility for the final interpretation in all cases; however, the study design does not permit direct assessment of the extent to which AI outputs influenced independent decision-making. This limitation is particularly relevant for inexperienced observers and should be considered when interpreting the observed improvements in concordance.

The educational implications of AI-assisted pathology tools remain an evolving area of investigation. Previous studies have suggested that AI-assisted platforms may facilitate learning through standardized visual guidance, quantitative feedback, and exposure to structured assessment criteria [32,33,34]. In the present study, AI-assisted assessment was associated with improved scoring consistency among medical students following limited introductory training. However, the current study was not designed to directly evaluate knowledge acquisition, long-term learning outcomes, or independent diagnostic competency. Accordingly, the findings should be interpreted as demonstrating improved observer concordance during AI-assisted evaluation rather than definitive evidence of educational efficacy.

Despite these encouraging findings, several limitations should be acknowledged. First, the study was conducted at a single institution with a relatively limited number of observers, which may restrict generalizability. Second, HER2-equivocal (2+) cases were not systematically confirmed by fluorescence in situ hybridization (FISH), potentially affecting classification benchmarking in selected cases. Third, although a washout interval was implemented to reduce recall bias, residual memory effects cannot be entirely excluded. The fixed sequence of assessment rounds (Visual Dx followed by AI-Assisted Dx) also does not permit a formal separation of AI-related improvement from learning or case-familiarity effects. We consider it unlikely, however, that observers retained case-specific recall of the 200 individual quantitative scores generated per round across this four-week interval; furthermore, the magnitude of improvement was not uniform across biomarkers and was not confined to novice observers, both of which argue against a purely mnemonic explanation. Nonetheless, a counterbalanced or randomized-order design remains warranted in future confirmatory studies to formally exclude this possibility. Finally, the study focused primarily on observer agreement metrics and did not assess workflow efficiency, diagnostic turnaround time, or long-term clinical impact.

Overall, our findings support the use of AI-assisted digital pathology tools as adjunctive systems that may improve scoring consistency and reduce variability in breast cancer biomarker assessment. Further multi-institutional studies involving larger observer cohorts, including an independent external validation cohort, and prospective clinical implementation settings are warranted to better define the role of AI-assisted workflows in both routine diagnostics and pathology education.

## 5. Conclusions

Accurate and reproducible assessment of predictive and prognostic biomarkers, including ER, PR, HER2, and Ki67, remains essential for therapeutic decision-making in breast cancer and for the broader implementation of precision oncology approaches. As treatment strategies become increasingly dependent on biomarker-driven patient stratification, there is a growing need for standardized and reproducible interpretation of immunohistochemical assays within routine diagnostic practice.

In this prospective observer study, AI-assisted assessment using HALO Breast IHC AI was associated with improved interobserver agreement and reduced scoring variability across multiple breast cancer biomarkers among both novice observers and experienced pathologists. The observed improvements were particularly evident for biomarkers known to demonstrate substantial interpretive variability, including HER2 and Ki67. These findings support the potential role of AI-assisted image analysis as an adjunctive decision-support tool within digital pathology workflows. As this study evaluated interobserver agreement rather than diagnostic accuracy or patient outcomes, conclusions regarding direct clinical implementation should be considered preliminary pending outcome-based validation.

Importantly, AI-assisted systems should be viewed as complementary to, rather than replacements for, expert pathological interpretation. When appropriately validated and integrated into clinical workflows, such tools may contribute to improved standardization, quality assurance, and reproducibility in biomarker assessment. In addition, AI-assisted platforms may hold potential value in training and educational contexts by facilitating structured and quantitative assessment approaches; however, as the present study was not designed to evaluate knowledge acquisition or learning outcomes directly, this potential application warrants dedicated evaluation in future studies before firmer conclusions can be drawn.

Further multi-institutional studies involving larger observer cohorts, including an independent external validation cohort, and prospective clinical implementation are warranted to better define the long-term clinical and educational impact of AI-assisted biomarker evaluation in breast cancer pathology.

## Figures and Tables

**Table 1:** Clinicopathological characteristics and biomarker distribution of the study cohort.

	Ductal		Lobular		Other	
**%cases (N)**	84% (42)		8% (4)		8% (4)	
	Positive			Negative		
**ER** (≥1% positivity threshold)	78% (39)			22% (11)		
**PR** (≥1% positivity threshold)	64% (32)			36% (18)		
**Ki67** (>15% positivity threshold)	42% (21)			58% (29)		
	0	1+		2+		3+
**HER2**	46% (23)	22% (11)		20% (10)		12% (6)

Fifty invasive breast carcinoma cases were sequentially selected from the archives of the Institute of Tissue Medicine and Pathology (ITMP), University of Bern. The cohort included a representative distribution of histological subtypes and biomarker expression profiles encountered in routine clinical practice. ER and PR positivity were defined according to ASCO/CAP guidelines using a ≥1% positivity threshold. Ki67 status was categorized using a >15% cutoff. HER2 scoring was classified according to standard clinical criteria (0–3+).

**Table 2:** Concordance and discordance between observer assessments, AI-assisted classifications, and routine clinical metadata.

Stain	Disagreement with Clinical Metadata	Complete Disagreement with Scorers	Complete Agreement with Scorers
ER	6% (3/50)	0% (0/50)	92% (46/50)
PR	10% (5/50)	0% (0/50)	92% (46/50)
Ki67	22% (11/50)	0% (0/50)	46% (23/50)
HER2	14% (7/49)	0% (0/49)	80% (39/49)

Percentage of cases demonstrating complete observer concordance (5/5 observers) with AI-assisted classifications or with routine clinical metadata across ER, PR, HER2, and Ki67 assessments. Discordant cases represent instances in which observer classifications differed from AI-assisted outputs or routine clinical assessment data.

**Table 3:** ICC analysis: intraclass correlation coefficients of scores from Visual Dx and AI-assisted Dx per biomarker.

	Visual Dx	AI-Assisted Dx
**ER**		
Students	0.76	0.95
Pathologists	0.97	1.00
Combined	0.83	0.97
**PR**		
Students	0.70	0.99
Pathologists	0.86	1.00
Combined	0.77	0.99
**Ki67**		
Students	0.78	0.99
Pathologists	0.74	1.00
Combined	0.76	0.99

**Table 4:** Fleiss’ kappa analysis.

	Visual Dx	AI-Assisted Dx
**ER**		
Students	0.64	0.80
Pathologists	0.94	1.00
Combined	0.74	0.88
**PR**		
Students	0.17	0.81
Pathologists	0.33	0.89
Combined	0.23	0.76
**Ki67**		
Students	0.18	0.92
Pathologists	0.58	1
Combined	0.30	0.95
**HER2**		
Students	0.44	0.78
Pathologists	0.46	1
Combined	0.42	0.79

Consensus agreement between scorers (Students, Pathologists, or Combined) from Visual Dx and AI-assisted Dx per biomarker. All Fleiss’ kappa estimates were significantly different from chance agreement (*p* < 0.001), except for Ki67 (Visual Dx, Students; *p* = 0.029).

**Table 5:** Mixed-effects modelling of categorical biomarker scores. Cumulative link mixed models were used to evaluate the effects of reader group (student versus pathologist), scoring round (visual versus AI-assisted), and their interaction on categorical scoring outcomes for ER, PR, Ki67, and HER2. Estimates (β), standard errors (SEs), and *p*-values are shown for fixed effects.

	Effect	Estimate (β)	SE	*p*-Value
**ER**	Student vs. Pathologist	−1.40	0.84	0.095
	AI-Assisted vs. Visual	−1.16	0.90	0.198
	Student × AI-Assisted	+1.40	1.14	0.220
**PR**	Student vs. Pathologist	+2.14	0.81	0.008
	AI-Assisted vs. Visual	+2.91	0.92	0.002
	Student × AI-Assisted	−2.91	1.13	0.010
**Ki67**	Student vs. Pathologist	+2.01	0.43	<0.001
	AI-Assisted vs. Visual	−0.19	0.44	0.666
	Student × AI-Assisted	−1.95	0.59	<0.001
**HER2**	Student vs. Pathologist	+2.97	0.36	<0.001
	AI-Assisted vs. Visual	+0.61	0.34	0.077
	Student × AI-Assisted	−2.10	0.46	<0.001

## Data Availability

The datasets generated and analyzed during the current study are not publicly available due to confidentiality considerations but are available from the corresponding author upon reasonable request.

## Supplementary Materials

The following supporting information can be downloaded at: https://www.mdpi.com/article/10.3390/cancers18183003/s1, Table S1: Number of slides excluded from analysis.

## Abbreviations

ER	estrogen receptor
PR	progesterone receptor
HER2	human epidermal growth factor receptor 2
ASCO/CAP	American Society of Clinical Oncology/College of American Pathologists
DCIS	ductal carcinoma in situ
ITMP	Institute of Tissue Medicine and Pathology, University of Bern
TRU	Translational Research Unit, University of Bern
ICC	intraclass correlation coefficient
