# Supplementary material for: AI-Assisted Scoring Improves Interobserver Agreement in Breast Cancer Biomarker Evaluation
Source: Cancers (Basel). 2026 Sep 16;18(18):3003. doi: 10.3390/cancers18183003 (PMC13604825; doi:10.3390/cancers18183003)
Supplement: Supplementary file 1 [file cancers-18-03003-s001.zip › cancers-4548561-supplementary.pdf]

Supplementary Materials

Supplementary Table S1. Number of slides excluded from analysis.

Slides were excluded from analysis due to a lack of scores provided by pathologists or students, combined across all observers within each group.

Biomarker	Agreement Metric	Visual Dx	AI-Assisted Dx
ER	ICC	3	3
ER	Fleiss' kappa	2	3
PR	ICC	4	4
PR	Fleiss' kappa	4	1
Ki67	ICC	4	0
Ki67	Fleiss' kappa	4	0
HER2	Fleiss' kappa	4	1

HER2 was assessed exclusively via categorical (ASCO/CAP) classification and was therefore not evaluated using ICC.
